# From the Editor's Desk

**Published:** 2016

**Authors:** PJ Commerford

**Affiliations:** Editor-in-Chief

This issue carries several important messages for those interested in cardiovascular heath in Africa. In the landmark Addis Ababa communiqué, Watkins and colleagues (page 184) describe seven essential actions aimed at eliminating rheumatic heart disease (RHD) in Africa. The distinguished group of authors are widely representative of Africans knowledgeable and active in both research and clinical service in this field and they are supported by international experts. Most importantly, this third All-Africa Workshop on Acute Rheumatic Fever and Rheumatic Heart Disease was hosted by the social cluster of the African Union Commission, and the communiqué has since been endorsed by African Union heads of state. Therefore the political will and support so necessary for successful implementation seems to be available and will be essential in the years ahead. The communiqué identifies that one of the barriers to eradicating RHD in Africa is that there are few centres capable of providing cardiac surgery, and action five aims to ‘Establish centres of excellence for cardiac surgery, which will sustainably deliver state-of-the-art surgical care, train the next generation of African cardiac practitioners, and conduct research on endemic cardiovascular diseases, including RHD’.

Against the background of that statement it is disturbing to read the position paper of the South African Heart Association by Sliwa and colleagues (page 188) on training in cardiology and cardiothoracic surgery in South Africa. The authors, all experienced in their fields of expertise and many are responsible for providing training in these fields, document the lack of training opportunities, the lack of adequate facilities and the failure of the state to expand and enlarge facilities so as to keep up with the population expansion. For the majority of South Africans who lack medical insurance or the funds to access private healthcare and who have heart disease, the situation has worsened over the last decade, with longer waiting lists for cardiac surgery at some tertiary centres than were seen previously, and a lack of simple monitoring tools such as echocardiography at many secondary level facilities. As the authors point out, clinicians have limited powers to alter the situation, and urgent action at government level is needed.

So we have the paradoxical situation where we publish two conflicting statements on the state of cardiovascular care in Africa. One is an encouraging, visionary message that the need for improved facilities for such care is recognised by both healthcare professionals and politicians, the other sketches the existing situation in South Africa where there has been no progression and in fact retrogression in provision of such care for the majority of the population. Which of the two scenarios play out in the future will depend on the continued involvement of the cardiovascular healthcare community and its active and successful interaction with politicians and governments.

An interesting review of research output in sub-Saharan Africa (SSA) and international collaboration in that research is provided by Ettarh (page 194). This study provides a picture over 10 years of the volume and scientific impact of international collaboration in cardiovascular research in SSA. This may be the first study of its kind and encouragingly demonstrates that research output is increasing and collaboration appears to be improving. Unfortunately, the extent of collaboration within SSA is very limited compared to the level of collaboration with other non-SSA countries. This pattern has been observed with data for all of the scientific output of the region. The potential benefits of increased collaboration in the region are described and should be an encouragement to junior researchers to widen collaboration in SSA.

Research into ethnic differences in risk factors for cardiac and other non-communicable diseases may provide insight into possible strategies to stem the predicted increase in these diseases in Africa. Keswell and co-authors (page 177) examined associations between body fat distribution, insulin resistance and dyslipidaemia in black and white South African women. The novel finding of this study was that central and peripheral fat depositions were independently associated with insulin resistance in both black and white women, and with triglyceride levels in the black women. By contrast, fasting glucose concentrations were associated with centralisation of body fat in black, but not white women, whereas total cholesterol and low-density lipoprotein cholesterol concentrations were associated with centralisation of body fat in white, but not black women.

Hypertrophic cardiomyopathy (HCM) was historically thought to be rare among Africans but as Ntusi and colleagues point out (page 152), recent echocardiographic studies from the continent have dispelled that myth. They examined a consecutive series of patients with HCM (30.2% black African), prospectively enrolled from a tertiary referral centre and characterised them clinically, echocardiographically and genetically. They found HCM to occur more in men, and with a younger age of onset. Major symptoms and complications were similar to those reported in North American, Middle Eastern and Asian studies. Known and novel disease-causing mutations were identified in the MYH7 and MYBPC3 genes, with a lower yield of mutation screening of about 30%, compared to the expected 40 to 70% found elsewhere. The mortality rate in this contemporary African HCM series was, however, higher than reported elsewhere, although comparable to age- and gender-matched members of the South African population. Survival was predicted by NYHA functional class at last clinic visit.

Balloon mitral valvuloplasty (BMV) revolutionised the management of many patients with rheumatic mitral stenosis, and the advent of the innovative Inoue balloon in the early eighties further popularised the technique, which was remarkably successful in alleviating symptoms and improving the mitral valve area. The method of selecting the size of balloon to use, based on the height of the patient, always seemed unusual, to say the least, but produced good results. Tastan and co-workers (page 147) describe the use of echocardiography to select balloon size and their results seem to indicate that this may be a more preferable method.

